# A germline *JAK2* exon12 mutation and a late somatic *CALR* mutation in a patient with essential thrombocythemia

**DOI:** 10.3389/fonc.2023.1265022

**Published:** 2024-01-04

**Authors:** Zhuanghui Hao, Juan Li, Feng Gao, Weixiao Ren, Xiaomei Lu, Jinyi Feng, Chen Zhang, Sicheng Bian, Juan Xie, Ming Luo, Jianmei Chang, Wanfang Yang, Ruixia Hou, Daniel Muteb Muyey, Jing Xu, Jiangxia Cui, Xiuhua Chen, Hongwei Wang

**Affiliations:** ^1^ Institute of Hematology, The Second Hospital of Shanxi Medical University, Taiyuan, China; ^2^ Institute of Genetics, Changzhi Maternal and Child Health Hospital, Changzhi, China; ^3^ Clinical Laboratory, The First Hospital of Shanxi Medical University, Taiyuan, China

**Keywords:** essential thrombocythemia, JAK2 exon12 mutation, CALR type1 mutation, distinct phenotype, myeloproliferative neoplasms

## Abstract

**Background:**

It has been discovered that Janus kinase 2 (*JAK2*) exon12 mutations lead to the polycythemia vera (PV) phenotype, while somatic mutations of calreticulin (*CALR*) are associated with essential thrombocythemia (ET) or primary myelofibrosis. In this article, we report a case of ET with coexistence of *JAK2* exon12 and *CALR* mutations. The objective of this study was to elucidate the pathogenicity mechanism of a *JAK2* exon12 mutation (*JAK2*N533S) and the role of the coexistence of mutations on the hematological phenotype.

**Methods:**

We designed a colony analysis of tumor cells obtained from this patient, and attempted to identify mutant genes using DNA from hair follicles. Mutation impairment prediction and conservative analysis were conducted to predict the mutation impairment and structure of *JAK2*N533S. In addition, we conducted a functional analysis of *JAK2*N533S by constructing Ba/F3 cell models.

**Results:**

Three distinct tumor subclones, namely *JAK2*N533S^het+^/*CALR*type1^het^ **^+^**, *JAK2*N533S^het+^/*CALR* ^wt^, and *JAK2*N533S^het+^/*CALR*type1^hom^ **^+^**, were identified from the 17 selected erythroid and 21 selected granulocyte colonies. The analysis of hair follicles yielded positive results for *JAK2*N533S. According to the bioinformatics analysis, *JAK2*N533S may exert only a minor effect on protein function. Functional studies showed that *JAK2*N533S did not have a significant effect on the proliferation of Ba/F3 cells in the absence of interleukin-3 (IL-3), similar to wild-type *JAK2*. Notably, there were no increased phosphorylation levels of *JAK2*-downstream signaling proteins, including signal transducer and activator of transcription 3 (STAT3) and STAT5, in Ba/F3 cells harboring the *JAK2*N533S.

**Conclusion:**

Our study revealed that the *JAK2*N533S^het+^/*CALR*type1^het+^ subclone was linked to a significant expansion advantage in this patient, indicating that it may contribute to the development of the ET phenotype. We further demonstrated that *JAK2*N533S, as a noncanonical *JAK2* exon12 mutation, is a germline mutation that may not exert an effect on cell proliferation and protein function. These results and the present body of available data imply that certain noncanonical *JAK2* mutations are not gain-of-function mutations leading to the development of myeloproliferative neoplasms.

## 1 Introduction

The classic Philadelphia-negative myeloproliferative neoplasms (MPN), which include polycythemia vera (PV), essential thrombocythemia (ET), and primary myelofibrosis, are a diverse group of clonal disorders characterized by the excessive production of mature cells in the peripheral blood (1–3). Somatic mutations in genes, such as V617F mutation in Janus kinase 2 (*JAK2*), *JAK2*V617F, *JAK2* exon12, calreticulin (*CALR*) exon9, or *MPL* exon10, exhibit a high prevalence (almost 90%) among patients with MPN, and are the underlying etiology of these disorders (4–8). Initially, researchers thought that these mutations were mutually exclusive in patients with MPN. However, a few cases of MPN associated with multiple driver mutations, most commonly the coexistence of *JAK2*V617F and *CALR* mutations, have been reported (9–12).

In this study, we present the case of a patient with ET who carries both *JAK2* exon12 mutation (*JAK2*N533S) and *CALR* mutation (*CALR*type1). Clinical observations revealed that *JAK2* exon12 mutations are found in PV (13). It is puzzling that this patient presents with a *JAK2* exon12 mutation and exhibits an ET phenotype, but lacks the characteristic PV phenotype. Additionally, the biological function of *JAK2*N533S, an atypical *JAK2* mutation, remains largely unexplored. Therefore, further investigation is warranted to elucidate the mechanism by which the coexistence of mutations contributes to the hematological phenotype in this case.

## 2 Materials and methods

### 2.1 Patient

The patient was diagnosed with MPN according to the 2016 World Health Organization criteria in the Second Hospital of Shanxi Medical University. Mutational investigation in this patient was performed using a DNA sample from fresh bone marrow or peripheral blood samples. Patient history and clinical data were extracted from the medical records. This patient provided written informed consent, and this study was conducted in accordance with the tenets stipulated in the Declaration of Helsinki.

### 2.2 Colony-forming assays

A peripheral blood sample of this patient was collected, and peripheral blood mononuclear cells were isolated. The cells were plated at a density of 1×10^5^cells/mL, which provided an optimal density for colony selection without the risk of contamination by neighboring colonies. Erythroid colony-forming units were cultured in methylcellulose-based medium (STEMCELL Technologies,VAN,CAN) containing additional 3 U/mL erythropoietin for 15 days. Granulocyte colony-forming units were incubated in methylcellulose-based medium containing an additional 100 ng/mL granulocyte colony-stimulating factor for 15 days. Erythroid and granulocyte colonies were selected, and DNA was extracted, amplified using a 2×STA Master Mix Kit (BBI Life Science,HK,China), and sequenced for *JAK2* exon12 (sense 5’TGGGCCGAAGTCTGA CCCTTT 3’ and antisense 5’ ACAGAGCGAACCAATGC 3’) and *CALR* exon9 (sense 5’ TGGGGCGTAACAAAGGTG AG 3’ and antisense 5’ TGAAAGTTC TCGAGTCTCA CAGA 3’). After purifying the polymerase chain reaction product, Sanger sequencing (The Beijing Genomics Institute,SZX,China) was used to identify mutation sites.

### 2.3 Bioinformatics analysis

We performed pathogenicity analyses for the novel missense variant using Mutation Taster score (https://www.mutationtster.org/ ), E-SNPs&GO (https://esnpsandgo.biocomp.unibo.it/), Polymorphism Phenotyping version 2 (PolyPhen2; http://genetics.bwh.harvard.edu/pph2/), and Sorting Intolerant From Tolerant (SIFT; https://sift.bii.a-star.edu.sg/). Alignment of homologous sequences of *JAK2* protein from various species was performed using the Unipro UGENE software (49.1version, http://ugene.net/) to assess the conservation of amino acid residues at different sites. Protein three-dimensional (3D) model of wild-type *JAK2* (*JAK2* ^wt^) was used the homodimeric JAK2 pseudokinase-protein tyrosine kinase (PK-PTK) model by JAK2 PK-PTK model (ma-evjj8). The five models of *JAK2*S533 was constructed based on the above ma-evjj8 by AlphaFold (https://alphafold.com/) and ColabFold (https://github.com/sokrypton/ColabFold) (14, 15). For further analysis, we used the *JAK2*S533 model with the highest per-residue confidence score (predicted local distance difference test) compared with the other models. The PyMOL system (4.5.0 vision) was used to visualize the results of the protein model. Detailed data of the model and score are provided in **Supplementary Material 1**.

### 2.4 Plasmid construction and lentiviral infection

Complementary DNAs (cDNAs) for human *JAK2*N533S and control genes (*JAK2* ^wt^, *JAK2* K539L, and *JAK2*N542-E543del) were synthesized by GenScript (NKG,CN). All cDNAs were cloned into a pCDH-CMV- MCS-EF1-CopGFP-Puro lentiviral-vector. Lentiviral particles were produced in 293T cells to infect Ba/F3 cells. The efficiency of gene transfer into Ba/F3 cells was assessed by green fluorescent protein (GFP) using laser confocal microscopy (Olympus, Tokyo, Japan) and flow cytometry (Beckman, CA,USA).

### 2.5 Cell culture and Cell Counting Kit-8 assay

Ba/F3 cells were cultured in RPMI 1640 medium (Gibco, USA) containing 15% fetal calf serum (Thermo Fisher Scientific,NK,USA) and 10 ng/mL interleukin-3 (PeproTech,NJ, USA). Cell viability assay was performed using a CCK8 Assay Kit (DOJINDO,kumamato, Japan). For the CCK8 assay, 3,000 cells were seeded in each well of a 96-well plate. Each cell line was cultured for 5 days in the absence of IL-3.

### 2.6 Western blotting

Ba/F3 cells were deprived of IL-3 for 2 days. Total protein was extracted, processed using radioimmunoprecipitation assay (RIPA) buffer (Thermo Fisher Scientific, NK,USA), and supplemented with protease and phosphatase inhibitor mixture tablets (Thermo Fisher Scientific,NK,USA). Western blotting was performed by Simple Western (Protein Simple Technology,SV, USA). Antibodies against signal transducer and activator of transcription 3 (STAT3), STAT5, phospho-STAT3 (pSTAT3), and pSTAT5 were obtained from Cell Signaling Technologies (MA,USA).

## 3 Results

### 3.1 Patient clinical characteristics

**Figure 1:**
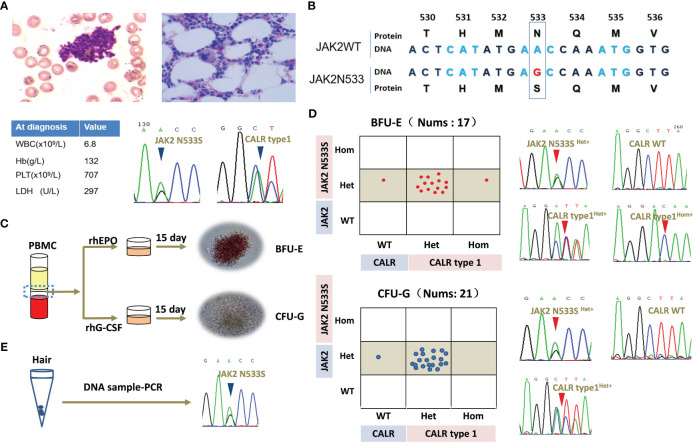
Clinical data of the patient and clonal evolution analysis for *JAK2*N533S and *CALR*type1. **(A)** Representative image of bone marrow biopsy and blood counts at diagnosis. Sanger Sequencing Charts of heterozygous *JAK2*N533S and *CALR*type1 using a peripheral blood samples at diagnosis. **(B)** Diagram of *JAK2*N533S. The substitution of base A with base G results in the change of asparagine to serine. **(C)** Schematic representation of erythroid and granulocyte colony-forming units. **(D)** Analysis of single colonies and sequencing chromatograms for *JAK2*N533S and *CALR*type1. **(E)** Sequencing chromatograms for *JAK2*N533S using DNA obtained from hair follicles. CALR, calreticulin; JAK2, Janus kinase 2.

A 75-year-old female patient presented with a gradual increase in platelet counts over 3 years. In December 2016, she was referred to the Second Hospital of Shanxi Medical University for evaluation of this marked thrombocytosis (**Figure 1A**). Despite the elevated platelet count, the patient was in good physical condition and did not experience any clinical symptoms, such as fever, itchy skin, facial flushing, bone pain, splenomegaly, or others. Furthermore, she did not have any complications, such as bleeding, thrombosis, or cerebrovascular disease.

A bone marrow biopsy was conducted to investigate the underlying reason for the thrombocytosis. The findings indicated normal cellularity with a normal ratio of myeloid to erythroid cells without fibrosis. However, there was a significant increase in megakaryopoiesis. Chromosomal analysis of 20 karyotypes revealed that the patient had a normal karyotype of 46, XX. Mutational analysis using peripheral blood revealed that the patient carried a *JAK2* exon12 mutation (*JAK2*N533S) and a *CALR* mutation (*CALR*type1). The patient was diagnosed with ET based on the platelet count (707 per mm^3^), proliferation mainly of the megakaryocytic lineage, and the presence of *CALR*type1. However, there was no evidence of PV apart from the presence of *JAK2*N533S. The patient’s blood routine returned to normal and remained stable after treatment with hydroxyurea. Currently, she remains in good health despite carrying *JAK2*N533S and *CALR*type1 (**Figures 1A, B**).

### 3.2 Clonal relationship of JAK2N533S and CALRtype1

The clonal relationship between *JAK2*N533S and *CALR*type1 was further investigated in this study. Peripheral blood mononuclear cells were isolated, and colony-forming assays were performed for erythroid cells and granulocytes. All 17 selected erythroid colonies exhibited a heterozygous *JAK2*N533S mutation (*JAK2*N533S^het^ **^+^**). Of those, 14 colonies (82%) also carried a heterozygous *CALR*type1 mutation (*CALR*type1^het+^), one colony (6%) harbored a homozygous *CALR*type1 mutation (*CALR*type1^hom+^), and two colonies (12%) exhibited a wild-type *CALR* (*CALR* ^wt^) genotype. In addition, all 21 selected granulocyte colonies exhibited a *JAK2*N533S^het+^. Among those, 20 colonies (95%) had a *CALR*type1^het+^, and one colony (5%) exhibited a *CALR* ^wt^ genotype (**Figures 1C, D**).

Because *JAK2N*533S^het+^ was present in all colonies, *JAK2*N533S was further tested using DNA extracted from hair follicles (**Figure 1E**). This analysis yielded positive results for the presence of *JAK2*N533S^het+^, indicating that this is a germline mutation. In contrast, among these colonies, 20 (95%) exhibited a *CALR*type1^het+^, while one (5%) had a *CALR* ^wt^ genotype. This evidence further indicated that *CALR*type1 was a late somatic event and occurred in a multipotent hematopoietic stem cell compartment capable of generating both myeloid and erythroid progeny.

Individual colony analysis revealed three distinct tumor subclones, namely *JAK2* N533S^het+^/*CALR*type1^het+^, *JAK2*N533S^het+^/*CALR* ^wt^, and *JAK2*N533S^het+^/*CALR*type1^hom+^. Notably, significant expansion was observed in the *JAK2*N533S^het+^/*CALR*type1^het+^ clone compared with the *JAK2*N533S^het+^/*CALR* ^wt^ clone. This finding suggested that the acquisition of *CALR* mutation provides a growth advantage over the presence of *JAK2*N533S alone (**Figures 1D, E**).

### 3.3 Mutation impairment prediction and conservative analysis

**Figure 2:**
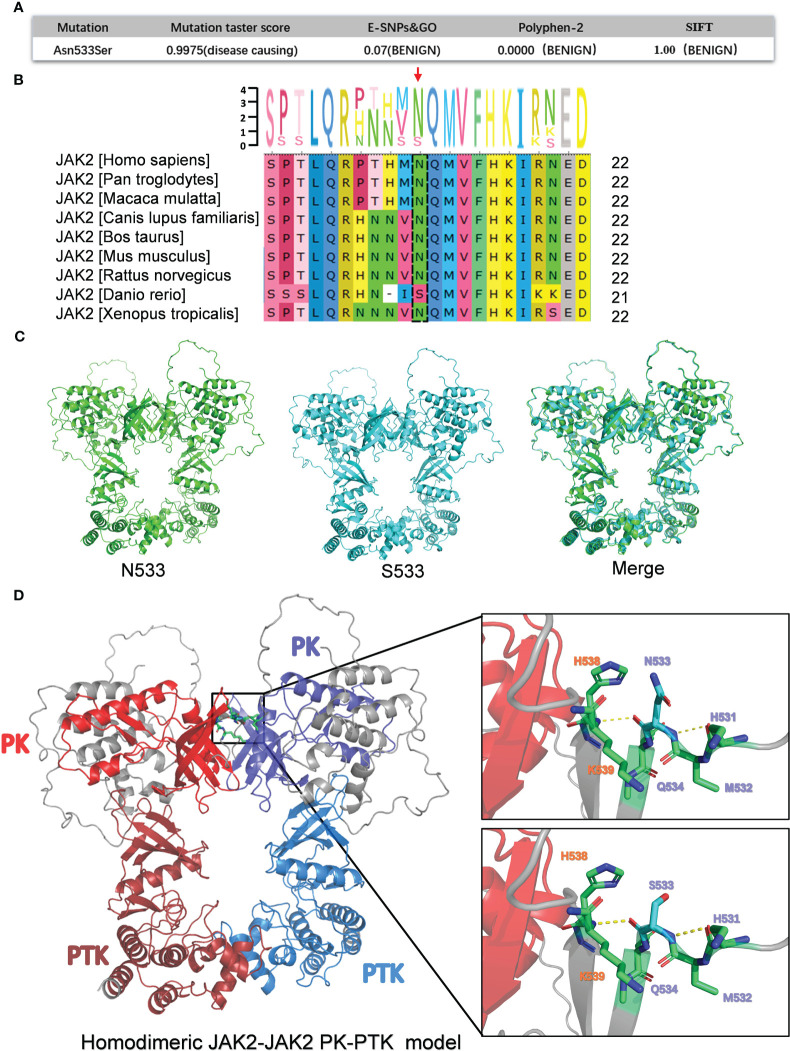
Bioinformatics analysis of the pathogenicity of *JAK2*N533S. **(A)** Prediction results for the pathogenicity of N533S based on the Mutation Taster score, E-SNPs&GO, PolyPhen-2, and SIFT. **(B)** Conservative analysis diagrams of the *JAK2* 533 site across different species. **(C)** Homodimeric *JAK2* PK-PTK model of *JAK2*N533 (green) and *JAK2*S533 (blue). **(D)** Left, homodimeric *JAK2* PK-PTK model of *JAK2*S533; the PK domain and PTK domain are marked using different colors. Right, zoom-in image showing the amino acids within 6 Å of N533 or S533. JAK2, Janus kinase 2; PK, pseudokinase; PTK, protein tyrosine kinase.

We sought to better understand the pathogenicity of *JAK2*N533S. Hence, a bioinformatics analysis was conducted to determine the function of *JAK2*N533S. In this analysis, various mutation impairment prediction tools were utilized to predict the impact of Asn533Ser substitution on the *JAK2* protein. The Mutation Taster score indicated that Asn533Ser substitution was pathogenic, suggesting a detrimental effect on protein function. However, other tools (e.g., E-SNPs&GO, PolyPhen-2, and SIFT) classified the N533S variant as benign, indicating that it is unlikely to significantly impact the protein function. The contradictory results obtained from the pathogenic prediction analysis of *JAK2*N533S are perplexing (**Figure 2A**). It is important to consider that different prediction methods may be characterized by inherent biases and limitations, which could contribute to discordant conclusions.

Additionally, a conservation analysis was performed to assess the level of conservation of Asn533 across different species. It was found that Asn533 is conserved in the *JAK2* gene among species, such as humans, *Pan troglodytes*, *Macaca mulatta*, etc. However, of note, in *Danio rerio*, Asn533 can naturally be replaced with Ser (**Figure 2B**).

Furthermore, a comparison of the three-dimensional model of the homodimeric JAK2 PK-PTK model containing N533 and S533 was conducted (**Figures 2C, D**). The S533 mutation does not appear to significantly alter the structure of the *JAK2* protein based on the merge of the JAK2 PK-PTK model of N533 and S533 (**Figure 2C**). The substitution of Asn with Ser could not trigger significant structural changes due to the slightly shorter length of Ser compared with Asn and their similar polar nature. Hence, it may cause subtle and confined alterations in *JAK2* structure.

Previous research has determined that the *JAK2*K539L mutation in *JAK2* exon12 is a pathogenic mutation that leads to *JAK2* constitutive activation. This activation occurs by disrupting the highly charged region of D620 and altering the salt bridge interaction between residues D620 and E621. Moreover, the change of salt bridging among Lys539, Glu592, and Glu596 in the periphery of the hydrophobic interface could potentially lead to the stabilization of a constitutive active dimer (15–18). Could the N533S mutation influence them by establishing or disrupting the surrounding salt bridging and hydrogen bonds to promote *JAK2* activation? There were no changes observed in the surrounding hydrogen bonds, and the N533S mutation did not establish a salt bridge interaction with residues D620, E621, and K539, as the distance between them exceeded 5 Å.

Collectively, based on the results of the bioinformatics analysis, *JAK2*N533S may exert only a minor effect on protein function. Further investigation and experimental studies are required to fully understand the functional consequences of this substitution.

### 3.4 Impact of JAK2N533S on Ba/F3 cell proliferation

**Figure 3:**
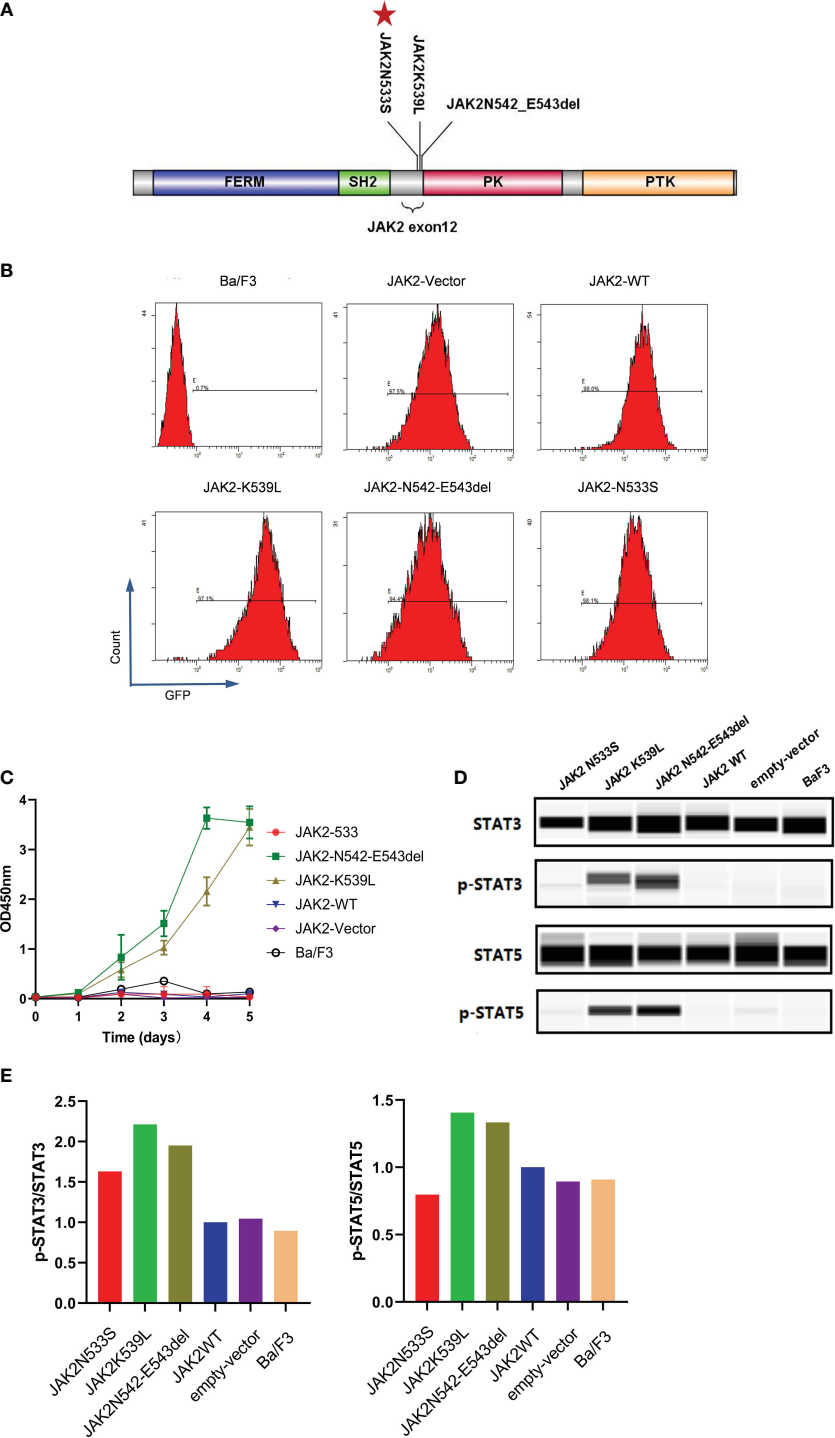
Functional analysis of *JAK2*N533S. **(A)** Schematic representation of the *JAK2* domain and *JAK2* exon12 mutations. Red stars in the panel indicate the positions of *JAK2*N533S. **(B)** Detection of GFP in different cell lines through flow cytometry. **(C)** IL-3-independent proliferation of *JAK2* mutation Ba/F3 models, detected by CCK8 assay. Data are presented as the mean ± standard deviation (SD) of three independent experiments; error bars indicate SD. **(D)** Lower phosphorylation levels of STAT3 and STAT3 in cells carrying *JAK2*N533S compared with cells harboring *JAK2*K539L and *JAK2*N542-E543del, detected by Simple Western. **(E)** Quantification of the expression of phospho-STAT3 (p-STAT3) and p-STAT5. Left, levels of p-STAT3 determined by densitometry of protein bands and normalized to those of STAT3. Right, levels of p-STAT5 determined by densitometry of protein bands and normalized to those of STAT5. CCK8, Cell Counting Kit-8; GFP, green fluorescent protein; IL-3, interleukin-3; STAT3/5, signal transducer and activator of transcription 3/5.

To investigate the functional effects of *JAK2*N533S, we cloned the cDNAs of *JAK2*N533S and control genes (*JAK2* ^wt^, *JAK2*K539L, and *JAK2*N542-E543del) into a lentiviral expression vector with an GFP and puromycin; of note, *JAK2*K539L and *JAK2*N542-E543del are the most common gain-of-function mutations in *JAK2* exon12 (**Figure 3A**). We used a lentiviral transduction to transfect the JAK2 gene with different mutation sites into Ba/F3 cells. We sorted the transgene-positive cells utilizing puromycin (2 ng/mL) and detected the positive rate of GFP by flow cytometry (**Figure 3B**). Next, we measured the IL-3-independent proliferation of cells. Each cell line was cultured for 5 days in the absence of IL-3. The experiment was conducted in triplicate. Cell viability assay was performed using CCK8. The control cells expressing *JAK2*K539L and *JAK2*N542-E543del showed significant accumulation, whereas there was no difference observed between cells carrying *JAK2*N533S and control cells expressing *JAK2* ^wt^, empty-vector, and untransfected Ba/F3 cells (**Figure 3C**). We further examined the levels of phosphorylated *JAK2*-downstream signal proteins, including STAT5 and STAT3. Cells were deprived of IL-3 cytokine for 2 days and analyzed by western blotting (ProteinSimple Technology) for phosphorylation of STAT5 and STAT3. Control cells carrying *JAK2*K539L and *JAK2*N542-E543del presented increased phosphorylation of STAT5(Tyr694) and STAT3(Tyr705), whereas those harboring *JAK2*N533S, *JAK2* ^wt^, empty-vector, and untransfected Ba/F3 cells did not show obvious activation of STAT5 and STAT3 (**Figures 3D, E**). The data obtained from the functional analysis indicated that *JAK2*N533S did not alter the function of *JAK2* protein.

## 4 Discussion

In this article, we have described a case with coexistence of *JAK2*N533S and *CALR*type1. Mutational analysis of a single colony allowed us to distinguish three distinct tumor subclones in this patient. Among these subclones, the *JAK2*N533S^het+^/*CALR*type1^het+^ clone had a significant expansion advantage over the other two clones. Thus, the *JAK2*N533S^het+^/*CALR*type1^het+^ clone was significantly associated with the ET phenotype of this patient. In contrast, we observed a small number of *JAK2*N533S^het+^/*CALR*type1^hom+^ subclones. It is unlikely that two independent mutation of *CALR* occurred at the exact same position (*CALR*type1). Therefore, we inferred that *CALR*type1^hom+^ could arise from the original *JAK2*N533S^het+^/*CALR*type1^het+^ clone by the loss of the *CALR* ^wt^ allele through deletion or uniparental disomy. The percentage of such homozygous colonies was very low. A possible reason is that loss of *CALR* ^wt^ may not provide a competitive advantage. In contrast, an alternative likely could be explained by the fact that the *CALR*type1^hom+^ subclone emerged shortly before the diagnosis. Therefore, the number of *JAK2*N533S^het+^/*CALR*type1^hom+^ clones was relatively low.

Furthermore, considering that only part of the selected colonies carried *CALR*type1, we inferred that *CALR*type1 is an acquired somatic mutation. All selected colonies carried *JAK2*N533S, indicating that this may be a germline mutation. We further tested this mutation using DNA samples obtained from hair follicles, indicating its potential emergence as a germline mutation. Regrettably, it was not possible to locate samples obtained from the parents of this patient. Hence, we were unable to strictly determine that *JAK2*N533S is a germline mutation.

According to the currently available data, *CALR*type1 is sufficient to induce the ET phenotype. Thus far, *JAK2* exon12 mutations have only been associated with the PV phenotype; *JAK2*K539L and *JAK2*N542-E543del are the most common gain-of-function mutations in *JAK2* exon12. In contrast, *JAK2*N533S (as a noncanonical *JAK2* exon12 mutation) has been rarely reported. In this investigation, we demonstrated that *JAK2*N533S did not exert an effect on the proliferation of cells through protein function prediction and cellular model function assays. The current data indicated that *JAK2* N533S did not contribute to MPN in this patient. Nevertheless, it is possible that this variant serves as a basis for *CALR* driver mutations. Moreover, the coexistence of *CALR*type1 and *JAK2*N533S could induce a stronger growth advantage to promote proliferation. Future research should focus on the effects of this coexistence.

In recent years, an increasing number of other noncanonical *JAK2* mutations have been identified in patients with MPN through next-generation sequencing (19–22). However, it has been observed that some of these *JAK2* mutations (e.g., G335D, F556W, Y590E,G571S, and Y613F) do not play a role in the pathogenesis of MPN (6, 20, 21, 23, 24). Our findings and other available data suggest that certain noncanonical *JAK2* mutations are not gain-of-function mutations leading to the development of MPN (1, 23–25). Therefore, it is recommended to assess the functional impact of noncanonical *JAK2* mutations in MPN cases at the time of diagnosis. This study on *JAK2*N533S can be used a reference in clinical practice.

## Data availability statement

The original contributions presented in the study are included in the article/**Supplementary Material**. Further inquiries can be directed to the corresponding authors.

## Ethics statement

The studies involving humans were approved by Ethics Committee of the Second Hospital of Shanxi Medical University. The studies were conducted in accordance with the local legislation and institutional requirements. The human samples used in this study were acquired from primarily isolated as part of your previous study for which ethical approval was obtained. Written informed consent for participation was not required from the participants or the participants’ legal guardians/next of kin in accordance with the national legislation and institutional requirements. The manuscript presents research on animals that do not require ethical approval for their study.

## Author contributions

ZH: Data curation, Formal analysis, Software, Writing – original draft, Writing – review & editing. JL: Formal analysis, Conceptualization, Writing – review & editing. FG: Investigation, Methodology, Conceptualization, Writing – review & editing. WR: Methodology, Writing – review & editing. XL: Methodology, Software, Writing – review & editing. JF: Methodology, Software, Writing – review & editing. CZ: Formal analysis, Investigation, Software, Writing – review & editing. SB: Methodology, Writing – review & editing. JXi: Methodology, Writing – review & editing. ML: Data curation, Methodology, Writing – review & editing. JMC: Data curation, Formal analysis, Methodology, Writing – review & editing. WY: Data curation, Formal analysis, Methodology, Writing – review & editing. RH: Data curation, Methodology, Validation, Writing – review & editing. DM: Writing – review & editing. JXu: Formal analysis, Methodology, Writing – review & editing. JXC: Funding acquisition, Investigation, Methodology, Writing – original draft, Writing – review & editing. XC: Data curation, Formal analysis, Funding acquisition, Investigation, Project administration, Supervision, Validation, Writing – original draft, Writing – review & editing. HW: Formal analysis, Funding acquisition, Methodology, Resources, Supervision, Validation, Writing – original draft, Writing – review & editing.
