# Supplementary figures and images for: A germline JAK2 exon12 mutation and a late somatic CALR mutation in a patient with essential thrombocythemia
Source: Front Oncol. 2024 Jan 4;13:1265022. doi: 10.3389/fonc.2023.1265022 (PMC10794477; doi:10.3389/fonc.2023.1265022)

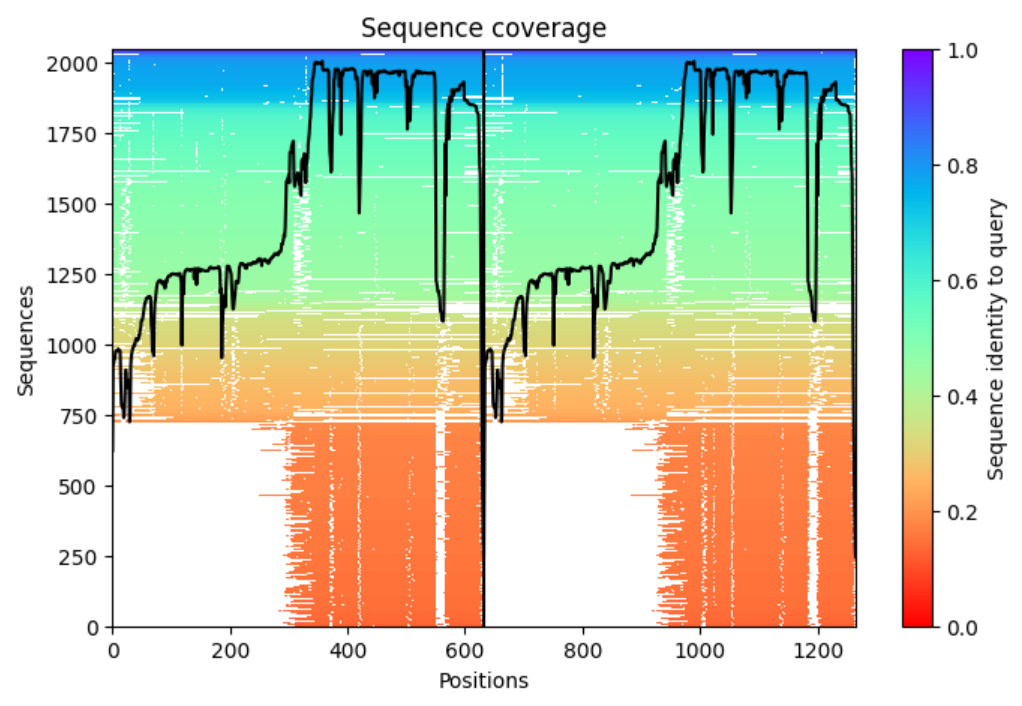

Supplement: Supplementary file 2 [file Image_1.png]

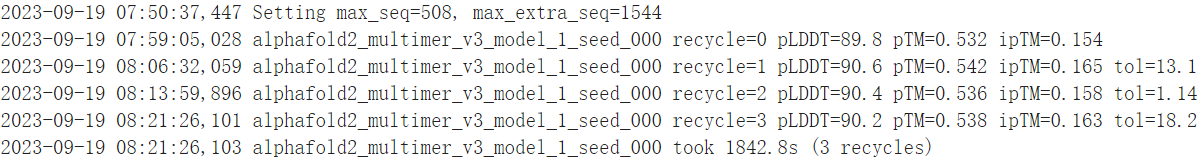

Supplement: Supplementary file 3 [file Image_2.png]

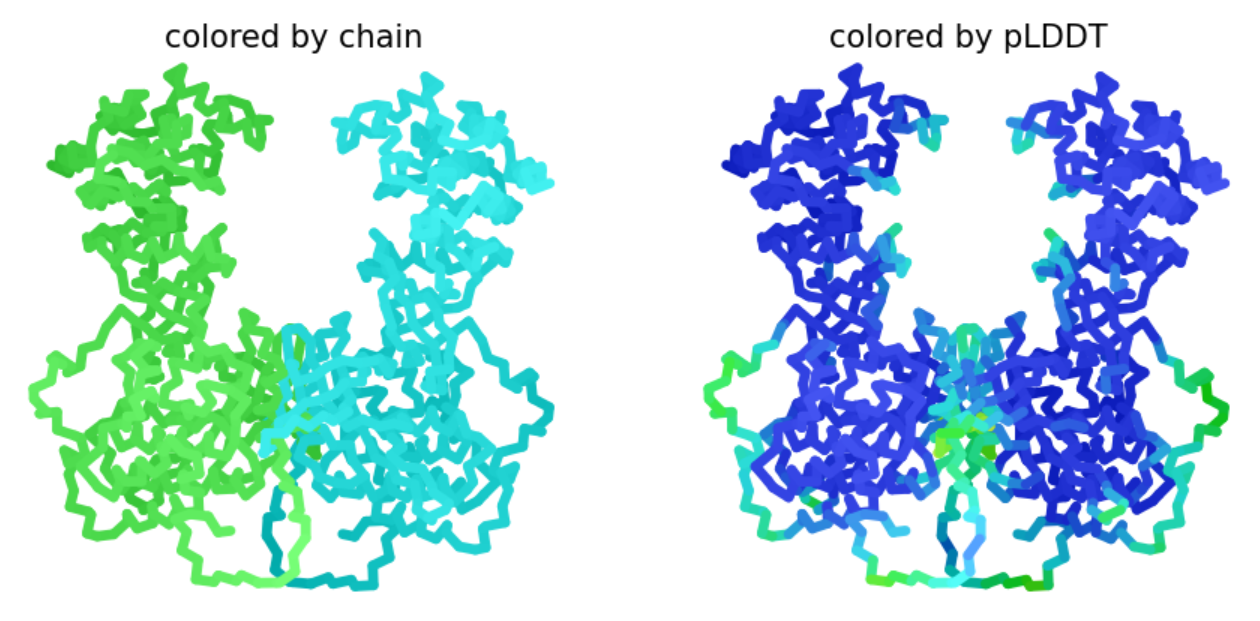

Supplement: Supplementary file 4 [file Image_3.png]

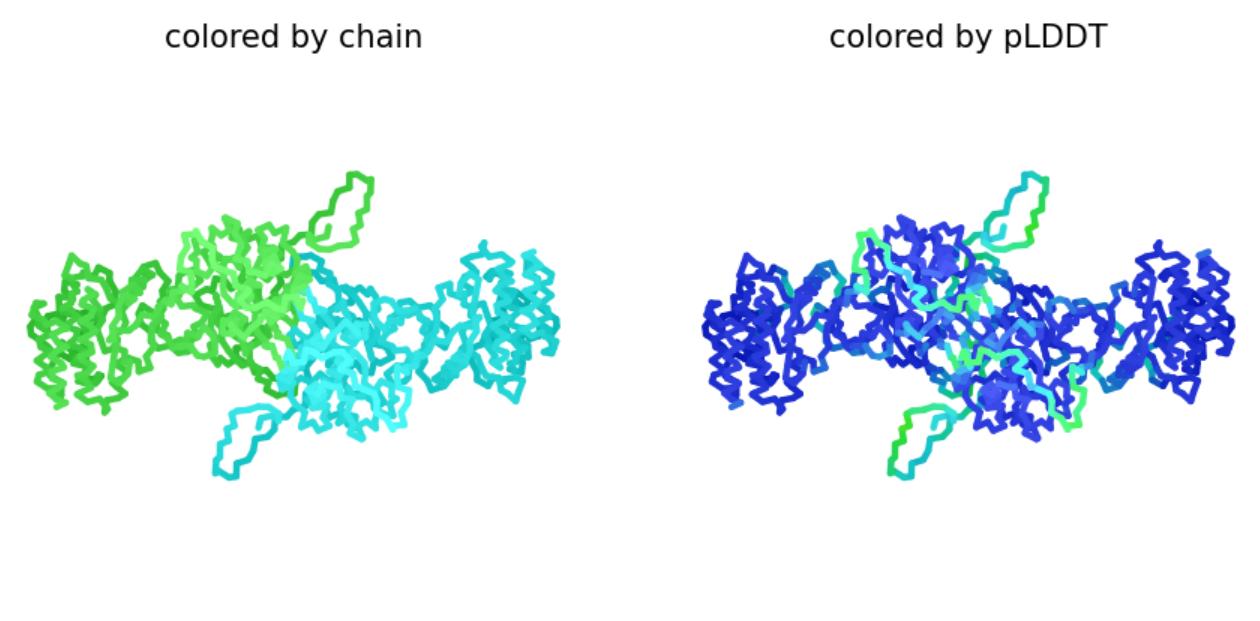

Supplement: Supplementary file 5 [file Image_4.png]

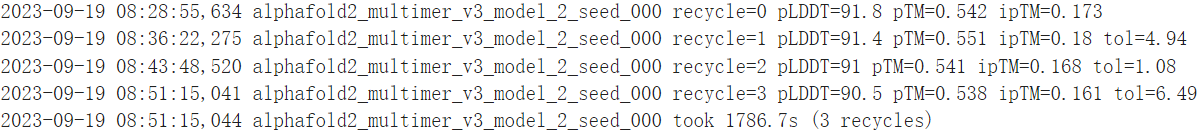

Supplement: Supplementary file 6 [file Image_5.png]

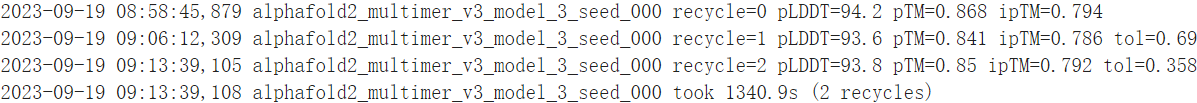

Supplement: Supplementary file 7 [file Image_6.png]

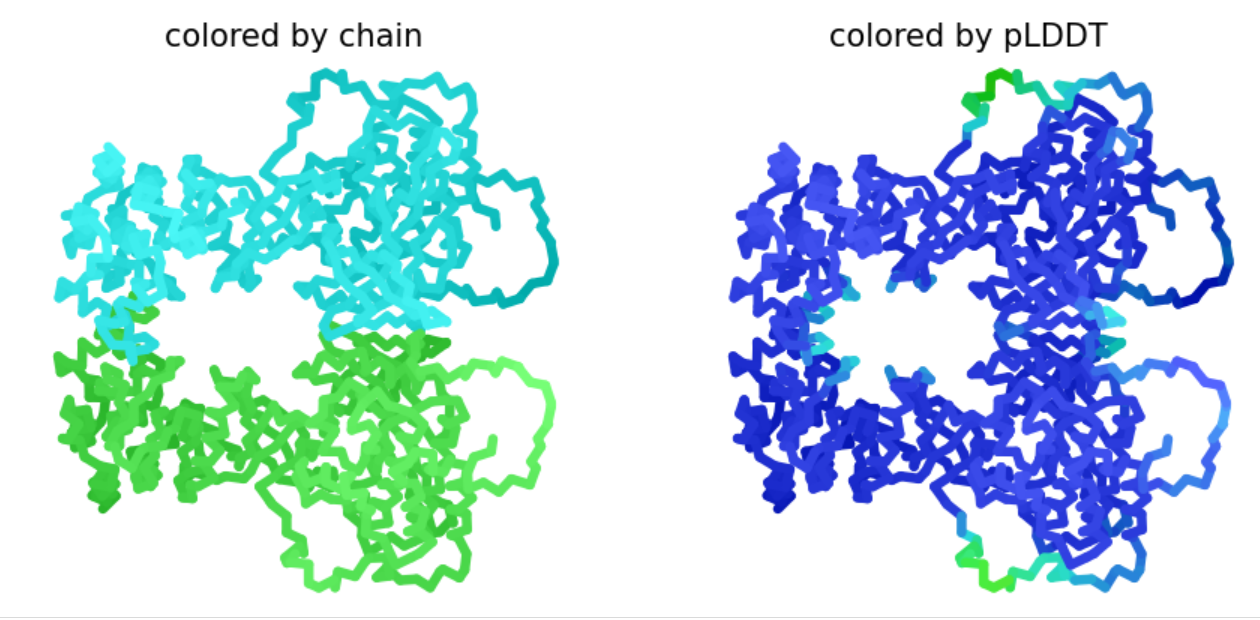

Supplement: Supplementary file 8 [file Image_7.png]

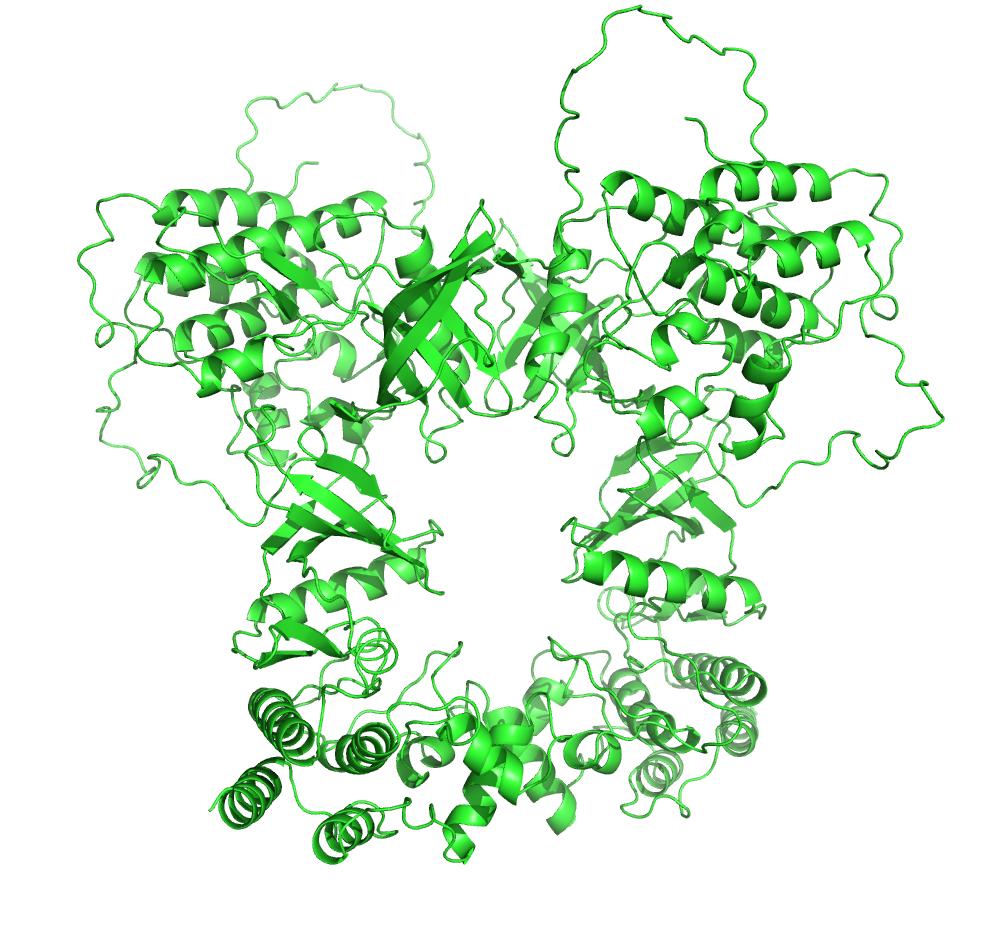

Supplement: Supplementary file 9 [file Image_8.png]

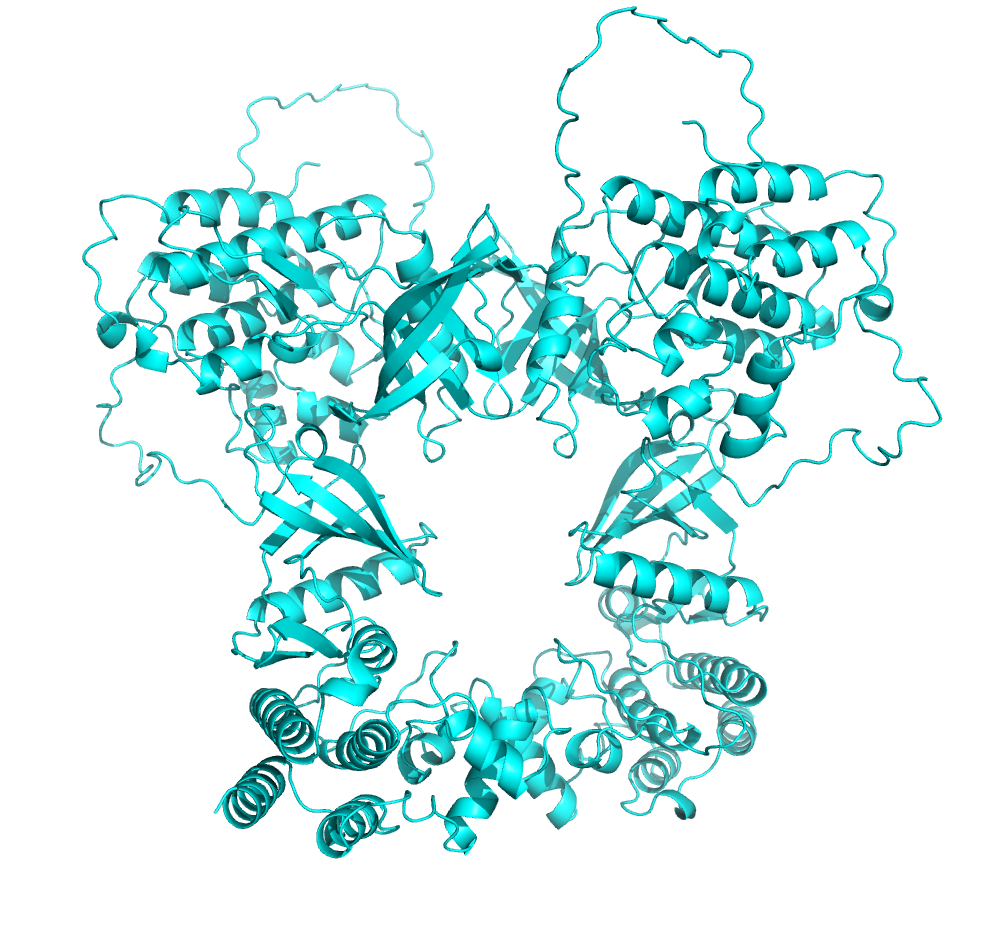

Supplement: Supplementary file 10 [file Image_9.png]

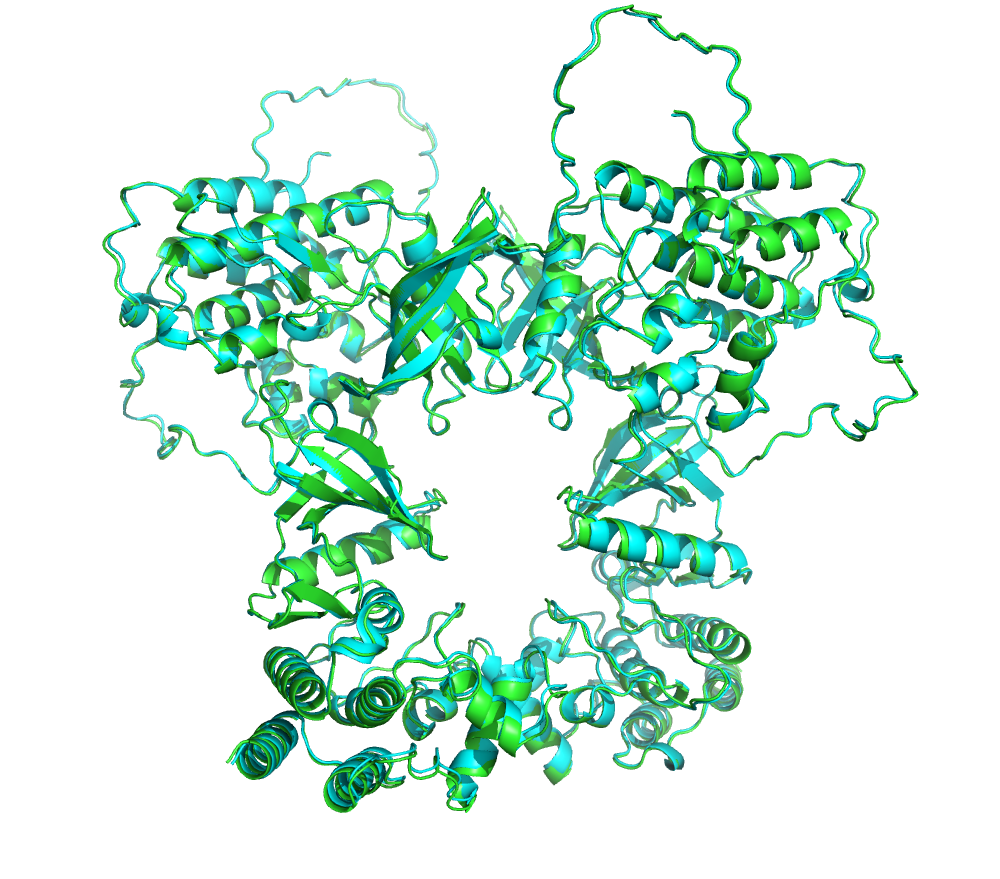

Supplement: Supplementary file 11 [file Image_10.png]

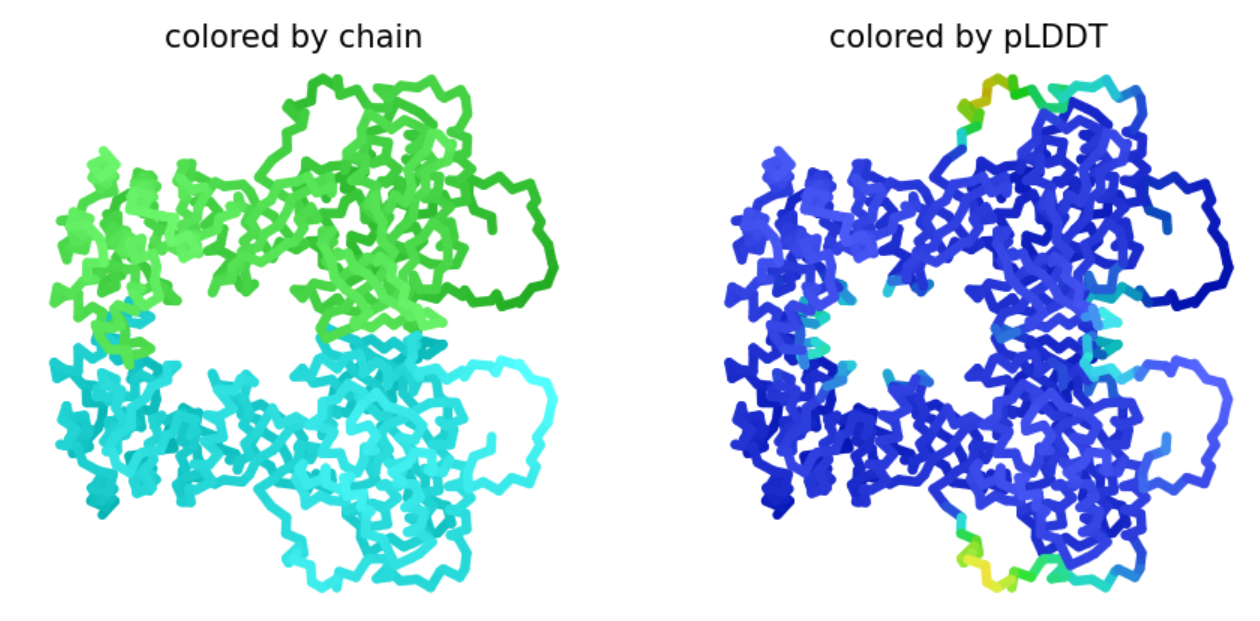

Supplement: Supplementary file 12 [file Image_11.png]

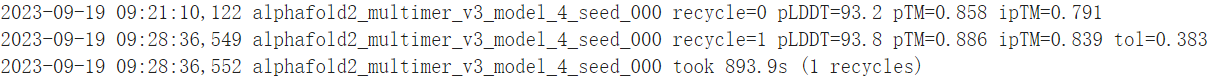

Supplement: Supplementary file 13 [file Image_12.png]

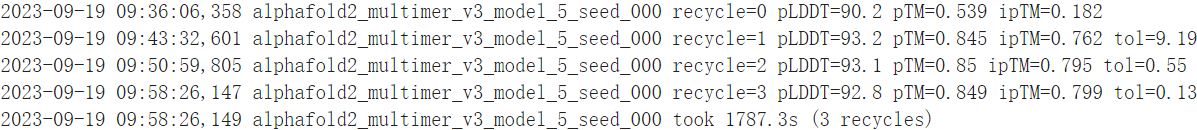

Supplement: Supplementary file 14 [file Image_13.png]

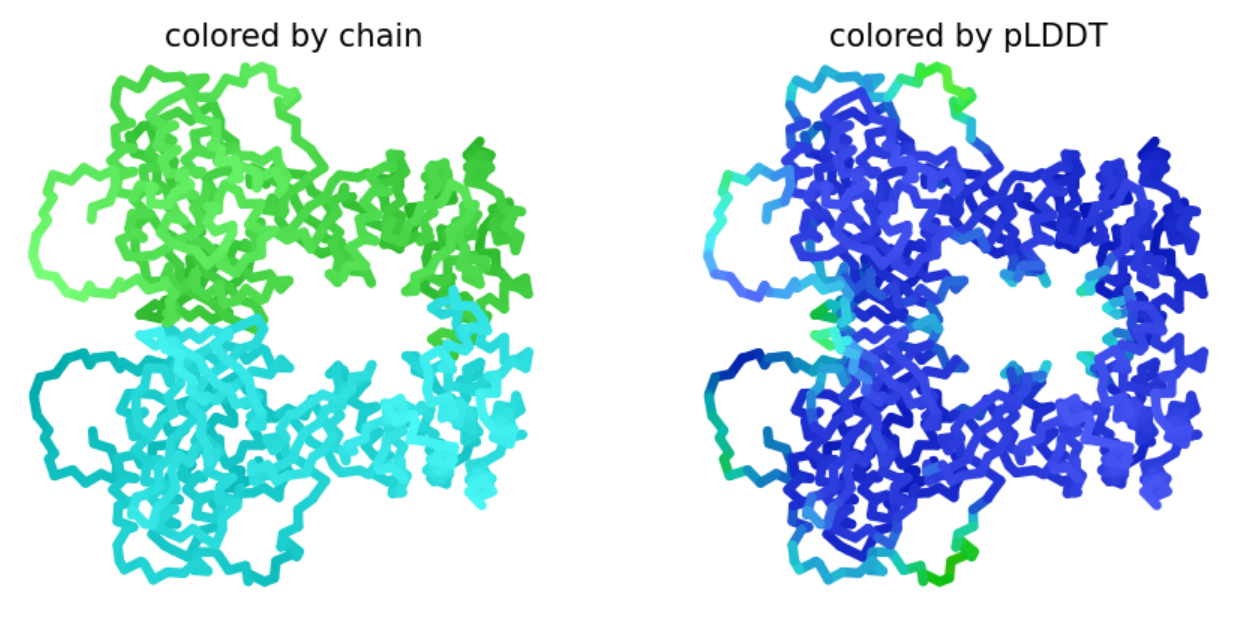

Supplement: Supplementary file 15 [file Image_14.png]
